# The 2007 Rift Valley Fever Outbreak in Sudan

**DOI:** 10.1371/journal.pntd.0001229

**Published:** 2011-09-27

**Authors:** Osama Ahmed Hassan, Clas Ahlm, Rosemary Sang, Magnus Evander

**Affiliations:** 1 Federal Ministry of Health, Khartoum, Sudan; 2 Department of Clinical Microbiology, Division of Infectious Diseases, Umeå University, Umeå, Sweden; 3 AVID project, Human Health Division, International Centre of Insect Physiology and Ecology, icipe – African Insect Science for Food and Health, Nairobi, Kenya; 4 Department of Clinical Microbiology, Division of Virology, Umeå University, Umeå, Sweden; London School of Hygiene & Tropical Medicine, United Kingdom

## Abstract

Rift Valley fever (RVF) is a neglected, emerging, mosquito-borne disease with severe negative impact on human and animal health and economy. RVF is caused by RVF virus (RVFV) affecting humans and a wide range of animals. The virus is transmitted through bites from mosquitoes and exposure to viremic blood, body fluids, or tissues of infected animals. During 2007 a large RVF outbreak occurred in Sudan with a total of 747 confirmed human cases including 230 deaths (case fatality 30.8%); although it has been estimated 75,000 were infected. It was most severe in White Nile, El Gezira, and Sennar states near to the White Nile and the Blue Nile Rivers. Notably, RVF was not demonstrated in livestock until after the human cases appeared and unfortunately, there are no records or reports of the number of affected animals or deaths. Ideally, animals should serve as sentinels to prevent loss of human life, but the situation here was reversed. Animal contact seemed to be the most dominant risk factor followed by animal products and mosquito bites. The Sudan outbreak followed an unusually heavy rainfall in the country with severe flooding and previous studies on RVF in Sudan suggest that RVFV is endemic in parts of Sudan. An RVF outbreak results in human disease, but also large economic loss with an impact beyond the immediate influence on the directly affected agricultural producers. The outbreak emphasizes the need for collaboration between veterinary and health authorities, entomologists, environmental specialists, and biologists, as the best strategy towards the prevention and control of RVF.

## Introduction

Globally, vector-borne diseases are responsible for almost 20% of the infectious diseases affecting humanity, and developing countries in Africa suffer most from the vector-borne disease burden and its socioeconomic consequences [1], [2]. Rift Valley fever (RVF), is a neglected, emerging, mosquito-borne disease with severe negative impact on human and animal health and economy, exaggerating poverty to already deprived communities. RVF is caused by RVF virus (RVFV) belonging to the *Bunyaviridae* family, genus *Phlebovirus* affecting humans and a wide range of animals [3], [4]. The virus is transmitted through bites from mosquitoes and exposure to blood, body fluids, or tissues of infected animals [3], [4]. RVFV was first isolated in Kenya 1930 [5], and 6 years later RVFV antibodies were found in human sera from southern Sudan [6]. Since then, RVF outbreaks have been recorded in many countries in sub-Saharan Africa [7], and recently it has emerged in new geographical areas with outbreaks reported in Yemen and Saudi Arabia in 2000 [8]. During 2006/2007 a large RVF outbreak occurred in the Horn of Africa. Sudan was severely affected during the second half of 2007 [9]. This article summarizes the knowledge gathered on RVFV activity in Sudan focusing on the 2007 outbreak and emphasizes the essential role of the policy maker in the prevention and control of RVF.

## Methods

Using the terms “Sudan” and “Rift Valley fever” 26 articles were identified by searching Medline through PubMed (http://www.ncbi.nlm.nih.gov/sites/entrez/) and by using “Rift Valley fever” alone, 1,010 articles were found. Additional articles were obtained by citation tracking of review and original articles. The books, reports, and fact sheets that include information about the disease were searched through the following sources:

World Health Organization (WHO) (http://www.who.int/); World Organization of Animal Health (OIE) (http://www.oie.int/); Food and Agriculture Organization of United Nation (FAO) (http://www.fao.org/); Google scholar (http://scholar.google.com/); United States Center for Emerging Issues (http://www.aphis.usda.gov/).

## The 2007 Outbreak in Sudan

The recent RVF outbreak in Sudan came to public attention October 18, 2007 when the Federal Ministry of Health (FMoH) Sudan asked the WHO to assist in the investigation and control of a suspected hemorrhagic fever outbreak (Table 1) [10]. The WHO and FMoH teams started investigations in the White Nile state on October 24, and on the basis of initial results, an outbreak of RVF was declared on October 28, and more help was requested for control measures (Table 1) [10]. In November an announcement was made regarding RVF in animals [11]. The first index human case was identified in early October [12], but it has been suggested that RVF cases appeared already in the beginning of September 2007 (Table 1) [13]–[15].

**Table 1:** Sequence of events, actions, and response related to the RVF outbreak in Sudan 2007.

Date	Event, Action, and/or Response
June 2007	Early warning alert [12]
June–August 2007	Heavy rains and flooding [46], [47]
September 2007	Suspected human RVF cases [13]–[15]
October 8–14, 2007	First human index case [12]
October 18, 2007	FMoH Sudan asks WHO for assistance [10]
October 24, 2007	FMoH and WHO teams start investigation [10], [11]
October 28, 2007	Outbreak of RVF declared [10], [11]
November 10, 2007	Outbreak of RVFV in livestock declared [11]
November 19, 2007	Start of targeted vaccination [73]
January 2008	End of outbreak [74]

**Figure 1:**
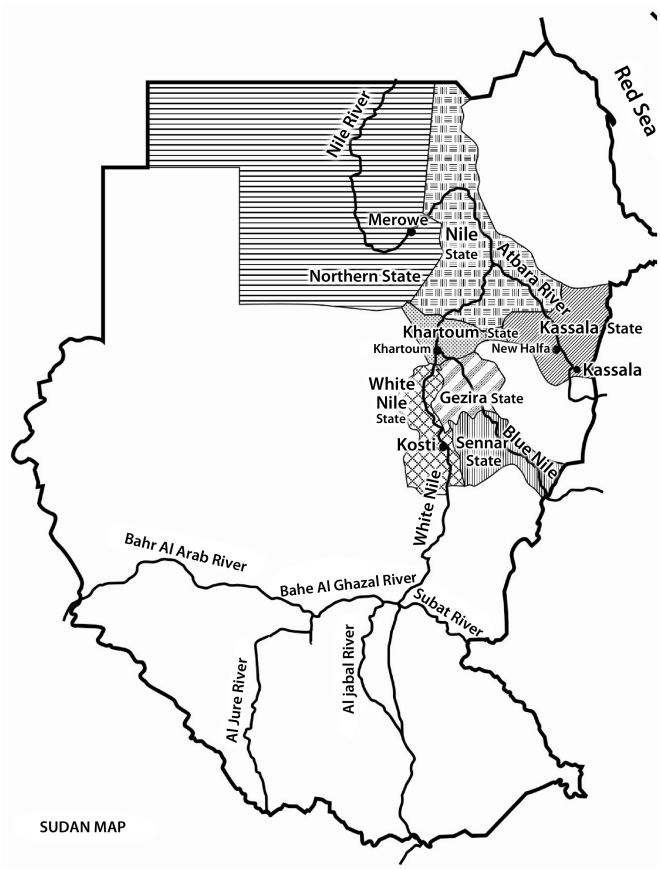
Map of Sudan 2007, showing the states reporting RVF during the outbreak.

At the end of the outbreak in January 2008, a total of 747 laboratory confirmed cases had been recorded with 230 deaths [16]. This epidemic was the first report of RVF since 1988/89 when RVFV was implicated in febrile illness outbreaks in Sudan [17], [18]. The 2007 outbreak was most severe in White Nile, El Gezira, and Sennar state near to the White Nile and the Blue Nile Rivers, the main branches of the Nile River in Sudan (Figure 1) [19]. Outbreaks of RVF were described during 1973, 1976, and 1981 [20]–[23], and serological surveys in several parts of Sudan have detected antibodies both in livestock [24], [25] and humans [17], [18], [23], [26]. RVF cases were also reported in 2007 from the states of Kassala, Khartoum, and River Nile, although the cases in Khartoum may have been acquired in other affected areas [9]. Furthermore, in New Halfa at Kassala, the heart of the second big irrigated agricultural scheme in the country, high RVFV immunoglobin G (IgG) antibody prevalence (82%) was found in patients with fever of unknown origin in 2007 [27]. Unfortunately no immunoglobin M (IgM) analysis was performed.

Sentinel animals had been surveyed during 2005–2006 in Kosti with no evidence of RVF [11] in the same area where RVF appeared in 2007 and 1973 [10], [20], [21]. After suspicion arose regarding the human outbreak, the joint investigation team started to collect new animal samples, and RVF was demonstrated in livestock followed by the declaration of an epizootic (Table 1). Unfortunately, there are no records or reports of the number of affected animals or deaths during the RVF outbreak [28]. Ideally, animals should serve as sentinels to prevent loss of human life, but the situation here was reversed, similar to the RVF outbreak in Kenya 2006–2007 [29]. Most probably, requisite levels of livestock infection are necessary before virus causes detectable severe disease in humans [29].

## Human Disease

Disabilities associated with the disease and the cost of treatment is a burden for the health system during an epidemic. In a recent publication the number of human cases estimated in the Sudan outbreak was 75,000 [12] compared to the 747 recorded cases from hospitals [16]. The underestimation of RVF is probably due to poor health infrastructure in rural areas. The high case fatality rate recorded (30.8%) in the epidemic was similar to some previous outbreaks in other countries [30], [31], but one can suspect that the high mortality was likely influenced by the low proportion of patients infected with RVF that seek medical care and were diagnosed. Although RVFV was isolated during the outbreak, there are no reports on viral genome comparison to other strains. Such sequence information could elucidate virus geographical transmission patterns and potentially important genetic differences that may exist. Interestingly, lineages isolated in Kenya differed in expansion and prevalence, suggesting important differences in reproductive fitness [32].

Clinical symptoms reported during the Sudan outbreak were fever, hemorrhagic symptoms, hepato-renal failure, and ocular complications [13]–[15]. Interestingly, 60% of RVF patients admitted to the Medani Hospital in El Gezira had renal impairment with high mortality (31%) [15], consistent with other studies from Sudan [14] and Saudi Arabia [33]. A recent report suggests an identifiable clinical syndrome of severe RVF, characterized by fever, large-joint arthralgia, and gastrointestinal complaints, later followed by jaundice, right upper-quadrant pain, and delirium, often coinciding with hemorrhagic manifestations [34]. The studies from Sudan suggest that renal failure could also be a possible identifying syndrome, an interesting feature similar to other *Bunyaviridae* such as the Hantaviruses that cause hemorrhagic fever with renal syndrome [35].

## Risk Factors for Severe RVFV Infection

Mosquitoes play a crucial role in transmission to animals and humans, but the most important risk factor for severe infection is contact with RVFV-infected animals such as consuming or handling products from sick animals, touching an aborted animal foetus, or being a herdsperson [36]. During the Sudan outbreak, animal contact seemed to be the most dominant risk factor followed by animal products and mosquito bites [15], similar to studies conducted during previous RVF outbreaks in other countries [37]–[40]. The findings concur with another study from Sudan stating that most of the animals such as sheep, cattle, goat, and camels stay very close to their owners' houses at night [13]. Many patients did not know how they contracted the disease, and one has to bear in mind that mosquito abundance was high in the area [15].

During the outbreak, a serious case of vertical transmission from mother to the baby was documented [41], similar to a previously reported case in Saudi Arabia in 2000 [42]. Many maternal deaths were observed in Central Sudan during the RVF outbreak [41], consistent with the claims recently made about the burden of emerging zoonotic infectious disease among women in general, and pregnant women in particular [43].

There was no evidence for horizontal transmission between humans in Sudan or elsewhere. However, health workers may contract many other viral diseases while dealing with patient excretions and therefore it is recommendable that the medical staffs take precautions with such cases. Laboratory personnel could also be at risk when handling samples [44].

## Sociodemographic Determinants

The majority of the RVF patients in Sudan lived in rural areas, but the reported number of shepherds infected was rather low, probably explained by less availability to health care leading to underestimation of true affected population [15]. As in Tanzania 2007 [45], more RVF cases were found in men than women, most probably due to participation of men in animal slaughter and breeding [13]–[15], [27]. Many of the cases among women were housewives, reflecting that women generally take care of the animals at home, prepare animal products and milk from potentially infected animals [13]. Most of the RVF patients were aged 15 to 29 years, younger than in Kenya [31] or Tanzania [45]. These were people in the active years of life [13], [15], resulting in an increased negative impact on the rural economy. The disease burden is most likely also increased when the infected person is the head of his/her household, but an in-depth study of the disease impact on the rural economy in Sudan is lacking. Interestingly, students were also infected most probably through mosquito bites [15], showing that the disease had spread not only to persons who deal with infected animals [13].

## Climatic and Geographical Conditions

Outbreaks of RVF are associated with heavy rainfall and flooding, increasing the population of mosquitoes leading to transmission of the infection, such as in the devastating outbreak in East Africa in 2006–2007 [31]. The Sudan outbreak also followed an unusually heavy rainfall in the country with severe flooding [46]. The heavy rains started in June 2007 and continued into August, causing an overflow of the main rivers. The areas where RVF outbreaks later occurred were among the flooded parts of Sudan and the 2007 season was one of the wettest recorded [47]. The Sudan outbreak was predicted in June 2007 using satellite measurements of sea surface temperatures, vegetation index, and elevated rainfall data (Table 1) [12]. Half of the human cases were geographically correctly predicted, and the cases in other areas were partly due to movements of viremic animals to other ecological zones, as in the El Gezira irrigation scheme [12].

The environment in the three affected states is suitable for agriculture with large numbers of susceptible animals serving as virus amplifiers. The humid climatic conditions support proliferation of the mosquitoes, the main vector of the disease. The El Gezira state was the worst affected area and most of the patients lived close to irrigation canal areas, linked to naturally occurring cycles involving livestock and mosquitoes [19], similar to the Ifakarra rice valley in Tanzania and highlands in Madagascar where large *Culex* mosquito populations could function as secondary vectors [12].

Modification of the environment has been associated with outbreaks of diseases transmitted by mosquitoes. For instance, water impoundments like large dams have been shown to exacerbate malaria transmission in malaria endemic parts of sub-Saharan Africa [48], similar to Egypt in 1977 when RVF appeared during the construction of Aswan dam on the Nile River [49]. Furthermore, an increased risk of RVFV amplification was reported from the Diama and Manantali Dams, implemented to regulate the Senegal River [50]. In 2009 Sudan finished construction of a new dam (Merowe dam) on the Nile river basin in the Northern state, 350 kilometers from the capital for the purpose of power and agriculture (Figure 1). Twenty years earlier there was human serological evidence of RVFV in the same area as the dam [17], suggesting virus circulation there. Surveillance among animals and mosquitoes around new dams and flooded areas could be of value as an early warning and has to be considered.

## The Role of Vectors and Possible Reservoirs

Mosquito species composition, densities, and infection varies within a country and different mosquito species could serve as epizootic/epidemic vectors of RVFV in diverse ecologies, creating a complex epidemiologic pattern [51]. Flood water *Aedes* species such as *Aedes mcintoshi*, *Ae. ochraceous*, *Ae. vexans* are considered the main mosquito vectors responsible for the vertical transmission of the virus [49], [52]–[54]. Infected eggs may stay for a long time in dry conditions until heavy rains fall and flood the grounds sufficiently to create good habitat and enable them to hatch and mature. Transovarially infected adult mosquitoes can then transmit RVFV to susceptible animals nearby, maintaining the virus during interepizootic period. High viremia in animals then leads to infection of secondary arthropod vector species including *Culex* and *Anopheles* species followed by spread to additional animals and humans [51], [53], [55]. Other insects like sand flies and ticks play some undetermined role.

In December 2007, adult and immature mosquitoes were collected in the White Nile state, one of the epidemic regions in Sudan, and Khartoum state, which supposedly only had imported cases [9]. The most frequent mosquito species with RVFV infection was *Anopheles gambiae arabiensis* in White Nile state, and *Culex pipiens* complex in Khartoum state [13]. Other species such as, *Aedes aegypti*, *An. coustani*, and *Cx. poicilipes* played a less important role. Since the vector collection was performed at the end of the outbreak the RVFV positive *Anopheles* and *Culex* mosquitoes were most probably secondary vectors considering previous reports [51], [53], [55]. Although RVF virus has been detected in a number of *Anopheles* species [4], [51], there is need to carry out vector competence studies to determine their role in the virus maintenance and transmission. In addition to threshold susceptibility to infection, the abundance, longevity, distribution, and feeding behavior must be considered in evaluating the epidemiological capacity of a species [4], [51]. RVFV was also detected in larvae of *Cx. pipens* complex, *An. gambiae arabiensis*, *An. coustani*, and *Ae. aegypti* and in male *An. gambiae arabiensis* mosquitoes [13]. There is no evidence for vertical transmission of RVFV in the *Anopheles* species, but immature mosquitoes have been shown to be infected by ingesting RVFV infected material [56].

As discussed above, infected mosquitoes were found 2007 in Khartoum [13]. Interestingly, during the 1973 epidemic a very small outbreak focus was reported in animals in the same area [24], suggesting that the virus was circulating around the capital. As in many other countries the big cities are growing and introduction of more livestock animals in the peripheral parts of the city may increase the chance of the disease occurrence. This might happen when the conditions are right, i.e., heavy rainfall, high competent mosquito and livestock populations [57].

Infected mosquitoes and animals are important in maintaining the level of virus during the interepidemic/epizootic periods. Small recurring local outbreaks most likely appear and in a recent retrospective study of RVF activity in Kenya, national RVF epizootics were recorded with an average interepizootic period of 3–6 years [57]. In Sudan, the 2007 outbreak took place 30 years after the last recorded outbreak. However, the population in certain areas had RVFV antibodies 20 years ago, suggesting that RVF is endemic in parts of Sudan [17], [18]. RVFV is infecting a multitude of animals and neutralizing antibodies have been found in African wildlife, e.g., buffalo, black rhino, lesser kudu, impala, and waterbuck [58], [59], which seem more resistant to disease than, e.g., sheep, cattle, and goats [55]. Donkeys, pigs, and rodents have also been discussed as potential reservoir for RVFV in Egypt [60], [61]. Since countries differ in livestock and wildlife species, climatic conditions, and mosquito populations, one must emphasize that the role of animals in the RVFV transmission cycle has to be put into context to aid better risk assessments in RVF contingency plans [57].

## Impact on Economy

Transmission of zoonotic diseases has increased worldwide in recent years because of increasingly intense livestock production in areas of proximity to human populations. An RVF outbreak results in human disease, but also large economic loss with an impact beyond the immediate influence on the directly affected agricultural producers [2], [62]. Livestock infection causes abortions and perinatal mortality (>95%) in herds (sheep, goats, cattle, and camels) used for meat and dairy production and income generation resulting in less food availability, and curtailed monetary income with major impact on poorer communities that do not have access to alternative sources of livelihood. Loss of earnings from livestock trade occasioned by export bans resulted in lack of finance for basic amenities like education, health, food, and shelter [2], [62]. Furthermore, inspection, monitoring, intervention, response, and financial assistance to affected producers are costly.

Livestock and livestock products are Sudan's second most important source of foreign exchange after oil. In 2000, after the RVF outbreak in the Arabian Peninsula, Saudi Arabia declared a ban on animal export from East Africa and the export from Sudan decreased significantly in 2000 and 2001 [63]. The economic effects of the 2007 outbreak have not been investigated, but one could suspect a huge economic impact on both the domestic and international animal market of the country. It is noteworthy that most of the exported animals come from the rural areas of Sudan, thus the ban has most severe consequences on the rural communities. Unfortunately no studies have been undertaken in Sudan to explore that. In Kenya, the 2007 RVF outbreak had wide-ranging impacts on the livestock sector as well as production impacts and employment losses (particularly for casual labor) and the estimated RVF induced losses were over US$32 million [64].

## Concluding Remarks

The RVF outbreak in Sudan was connected to heavy rainfall, flooding, and increased mosquito breeding and was first reported in humans. The lesson from Sudan makes it clear that human health is the result of sustainable relationships between humans, animals, and the environment, summarized in the new concept “One Health One World” [65]. Monitoring of animal disease and entomological events together with prediction models for climatic changes within risk areas should be used as indicators of an RVF outbreak [66]. Decision support tools have been developed [67], but a combined effort to study models of disease prevention and control before and during RVF outbreaks is needed.

**Key Learning Points**

- Rift Valley fever (RVF) is an important emerging viral zoonosis that causes disease both in animals and humans, and outbreaks of RVF are correlated to abundance of mosquitoes due to increased rainfall and flooding.
- The 2007 outbreak was the first reported RVF outbreak in Sudan for 36 years, although studies indicate that the virus has been circulating in Sudan for a long time.
- In the Sudan outbreak, humans, rather than livestock, served as sentinels. Surveillance could be improved by using animals as sentinels to prevent loss of human life.
- A One Health One World perspective is clearly needed for prevention and control of RVF. The capacity of veterinary authorities and entomologists should be enhanced to implement early warning systems.
- There is a need to carry out vector competence studies to determine the epidemiological capacity of different mosquito species and their role in the virus maintenance and transmission.

**Key Papers**

- Daubney R, Hudson JR (1931) Enzootic hepatitis or Rift Valley fever. An undescribed virus disease of sheep, cattle and man from East Africa. J Pathol Bacteriol 34: 545–579.
- Eisa M, Obeid HMA, El Sawi ASA (1977) Rift Valley fever in the Sudan. I. Results on field investigations of the first epizootic in Kosti District, 1973 Bull Anim Health Prod Afr 24: 343–347.
- Wilson ML (1994) Rift Valley fever virus ecology and the epidemiology of disease emergence. Ann N Y Acad Sci 740:169–180.
- Nguku PM, Sharif SK, Mutonga D, Amwayi S, Omolo J, et al. (2010) An investigation of a major outbreak of Rift Valley fever in Kenya: 2006–2007. Am J Trop Med Hyg 83: 5–13.
- Anyamba A, Linthicum KJ, Small J, Britch SC, Pak E, et al. (2010) Prediction, assessment of the Rift Valley fever activity in East and Southern Africa 2006–2008 and possible vector control strategies. Am J Trop Med Hyg 83: 43–51.

**Main Challenges**

- The development of RVF early warning systems and surveillance on an applicable regional level is of utmost importance, but policy makers have to be convinced by research and facts to understand that it is achievable and worthwhile.
- Lack of a One Health approach exacerbates RVF outbreaks in resource poor regions. Collaboration between veterinarians, entomologists, physicians, and environmental specialists must be encouraged to facilitate better surveillance.
- Capacity building and training is needed to improve recognition of human and animal cases and set-up of diagnostic methods.
- Livestock trade is the backbone of the rural economy and embargos could have devastating economic effects. Disease notification has to be encouraged, for instance by international collaboration and help with compensation to prevent further spread.
- No antiviral or human RVFV vaccine exist and the use of animal vaccines during outbreaks have been questioned [66], [68], [69]. The present vaccine can cause abortion [69] and improved vaccines using other strategies may be a future possibility [70]–[72]. The vaccine is often not delivered in time and most probably preventive vaccination would be more effective than vaccination in the beginning of an outbreak [66], [73]. One has to bear in mind that RVF can be transmitted among the animal herd because of use of multidose vaccination vials and reuse of needles and syringes [68].

## References

[1] LaBeaud AD (2008). Why arboviruses can be neglected tropical diseases.. PLoS Negl Trop Dis.

[2] Hotez PJ, Kamath A (2009). Neglected tropical diseases in sub-saharan Africa: review of their prevalence, distribution, and disease burden.. PLoS Negl Trop Dis.

[3] Flick R, Bouloy M (2005). Rift Valley fever virus.. Curr Mol Med.

[4] Pepin M, Bouloy M, Bird BH, Kemp A, Paweska J (2010). Rift Valley fever virus (Bunyaviridae: Phlebovirus): an update on pathogenesis, molecular epidemiology, vectors, diagnostics and prevention.. Vet Res.

[5] Daubney R, Hudson JR (1931). Enzootic hepatitis or Rift Valley fever. An undescribed virus disease of sheep, cattle and man from East Africa.. J Pathol Bacteriol.

[6] Findlay GJ, Stefanopoulo GM, Mac Callum F (1936). Présence d'anticorps contre la fièvre de la vallée du Rift dans le sang des africains.. Bull Soc Pathol Exot.

[7] Davies FG (2010). The historical and recent impact of Rift Valley fever in Africa.. Am J Trop Med Hyg.

[8] Balkhy HH, Memish ZA (2003). Rift Valley fever: an uninvited zoonosis in the Arabian peninsula.. Int J Antimicrob Agents.

[9] WHO (2007). Rift Valley fever in Sudan - update 4, December 21. Global Alert and Response.

[10] WHO (2007). Rift Valley fever in Sudan - update, November 7. Global Alert and Response.

[11] Garang GD (2007). A press release on Rift Valley Fever disease in Sudan 10/11/2007.

[12] Anyamba A, Linthicum KJ, Small J, Britch SC, Pak E (2010). Prediction, assessment of the Rift Valley fever activity in East and Southern Africa 2006–2008 and possible vector control strategies.. Am J Trop Med Hyg.

[13] Seufi AM, Galal FH (2010). Role of Culex and Anopheles mosquito species as potential vectors of rift valley fever virus in Sudan outbreak, 2007.. BMC Infect Dis.

[14] Adam AA, Karsany MS, Adam I (2010). Manifestations of severe Rift Valley fever in Sudan.. Int J Infect Dis.

[15] El Imam M, El Sabiq M, Omran M, Abdelkareem A, El Gaili Mohamed MA (2009). Acute renal failure associated with the Rift Valley Fever: a single center study.. Saudi J Kidney Dis Transpl.

[16] WHO (2008). Together for better health. A report on WHO collaborative programmes with the Government of Sudan and partners 2006–2007. WHO Sudan Biennial Report 2006–2007.

[17] Watts DM, el-Tigani A, Botros BA, Salib AW, Olson JG (1994). Arthropod-borne viral infections associated with a fever outbreak in the northern province of Sudan.. J Trop Med Hyg.

[18] McCarthy MC, Haberberger RL, Salib AW, Soliman BA, El-Tigani A (1996). Evaluation of arthropod-borne viruses and other infectious disease pathogens as the causes of febrile illnesses in the Khartoum Province of Sudan.. J Med Virol.

[19] Cooke FJ, Shapiro DS (2007). Rift Valley Fever in Sudan.. Int J Inf Dis.

[20] Eisa M, Obeid HMA, El Sawi ASA (1977). Rift Valley fever in the Sudan.. I. Results on field investigations of the first epizootic in Kosti District, 1973 Bull Anim Health Prod Afr.

[21] Eisa M, Obeid HMA (1977). Rift Valley fever in the Sudan. II. Isolation and identification of the virus from a recent epizootic in Kosti District, 1973.. Bull Anim Health Prod Afr.

[22] Eisa M, Kheir el-Sid ED, Shomein AM, Meegan JM (1980). An outbreak of Rift Valley fever in the Sudan–1976.. Trans R Soc Trop Med Hyg.

[23] Saleh AS, Mohammed KA, Hassan MM, Bucci TJ, Meegan JM (1981). Antibodies to Rift Valley fever virus in the human population of Sudan.. Trans R Soc Trop Med Hyg.

[24] Eisa M (1984). Preliminary survey of domestic animals of the Sudan for precipitating antibodies to Rift Valley fever virus.. J Hyg (Lond).

[25] Davies FG (1990). Rift Valley fever in the Sudan.. Trans R Soc Trop Med Hyg.

[26] Woodruff PW, Morrill JC, Burans JP, Hyams KC, Woody JN (1988). A study of viral and rickettsial exposure and causes of fever in Juba, southern Sudan.. Trans R Soc Trop Med Hyg.

[27] Hassanain AM, Noureldien W, Karsany MS, Saeed el NS, Aradaib IE (2010). Rift Valley Fever among febrile patients at New Halfa hospital, eastern Sudan.. Virol J.

[28] OIE (2008).

[29] Munyua P, Murithi RM, Wainwright S, Githinji J, Hightower A (2010). Rift Valley fever outbreak in livestock in Kenya, 2006–2007.. Am J Trop Med Hyg.

[30] Madani TA, Al-Mazrou YY, Al-Jeffri MH, Mishkhas AA, Al-Rabeah AM (2003). Rift Valley fever epidemic in Saudi Arabia: epidemiological, clinical, and laboratory characteristics.. Clin Infect Dis.

[31] Nguku PM, Sharif SK, Mutonga D, Amwayi S, Omolo J (2010). An investigation of a major outbreak of Rift Valley fever in Kenya: 2006–2007.. Am J Trop Med Hyg.

[32] Bird BH, Githinji JW, Macharia JM, Kasiiti JL, Muriithi RM (2008). Multiple virus lineages sharing recent common ancestry were associated with a Large Rift Valley fever outbreak among livestock in Kenya during 2006–2007.. J Virol.

[33] Al-Hazmi M, Ayoola EA, Abdurahman M, Banzal S, Ashraf J (2003). Epidemic Rift Valley fever in Saudi Arabia: a clinical study of severe illness in humans.. Clin Infect Dis.

[34] Kahlon SS, Peters CJ, Leduc J, Muchiri EM, Muiruri S (2010). Severe Rift Valley fever may present with a characteristic clinical syndrome.. Am J Trop Med Hyg.

[35] Pettersson L, Boman J, Juto P, Evander M, Ahlm C (2008). Outbreak of Puumala virus infection, Sweden.. Emerg Infect Dis.

[36] Anyangu AS, Gould LH, Sharif SK, Nguku PM, Omolo JO (2010). Risk factors for severe Rift Valley fever infection in Kenya, 2007.. Am J Trop Med Hyg.

[37] Brown JL, Dominik JW, Morrissey RL (1981). Respiratory infectivity of a recently isolated Egyptian strain of Rift Valley fever virus.. Infect Immun.

[38] Chambers PG, Swanepoel R (1980). Rift valley fever in abattoir workers.. Cent Afr J Med.

[39] McIntosh BM, Russell D, dos Santos I, Gear JH (1980). Rift Valley fever in humans in South Africa.. S Afr Med J.

[40] Woods CW, Karpati AM, Grein T, McCarthy N, Gaturuku P (2002). An outbreak of Rift Valley fever in Northeastern Kenya, 1997–98.. Emerg Infect Dis.

[41] Adam I, Karsany MS (2008). Case report: Rift Valley Fever with vertical transmission in a pregnant Sudanese woman.. J Med Virol.

[42] Arishi HM, Aqeel AY, Al Hazmi MM (2006). Vertical transmission of fatal Rift Valley fever in a newborn.. Ann Trop Paediatr.

[43] Theiler RN, Rasmussen SA, Treadwell TA, Jamieson DJ (2008). Emerging and zoonotic infections in women.. Infect Dis Clin North Am.

[44] WHO (2008). Rift Valley fever fact sheet.. Weekly epidemiological record.

[45] Mohamed M, Mosha F, Mghamba J, Zaki SR, Shieh WJ (2010). Epidemiologic and clinical aspects of a Rift Valley fever outbreak in humans in Tanzania, 2007.. Am J Trop Med Hyg.

[46] Moszynski P (2007). Flooding worsens in Sudan.. BMJ.

[47] FAO/GIEWS (2007). Floods cause damage in parts of several East African countries. Food and Agriculture Organization of the United Nations/Global Information and Early Warning System. Global Watch, 5 September 2007.

[48] Lautze J, McCartney M, Kirshen P, Olana D, Jayasinghe G (2007). Effect of a large dam on malaria risk: the Koka reservoir in Ethiopia.. Trop Med Int Health.

[49] Wilson ML (1994). Rift Valley fever virus ecology and the epidemiology of disease emergence.. Ann N Y Acad Sci.

[50] Digoutte JP, Peters CJ (1989). General aspects of the 1987 Rift Valley fever epidemic in Mauritania.. Res Virol.

[51] Sang R, Kioko E, Lutomiah J, Warigia M, Ochieng C (2010). Rift Valley fever virus epidemic in Kenya, 2006/2007: the entomologic investigations.. Am J Trop Med Hyg.

[52] Gerdes GH (2004). Rift Valley fever.. Rev Sci Tech.

[53] Davies FG, Linthicum KJ, James AD (1985). Rainfall and epizootic Rift Valley fever.. Bull World Health Organ.

[54] Linthicum KJ, Davies FG, Kairo A, Bailey CL (1985). Rift Valley fever virus (family Bunyaviridae, genus Phlebovirus). Isolations from Diptera collected during an inter-epizootic period in Kenya.. J Hyg (Lond).

[55] Davies FG (1975). Observations on the epidemiology of Rift Valley fever in Kenya.. J Hyg (Lond).

[56] Turell MJ, Linthicum KJ, Beaman JR (1990). Transmission of Rift-Valley fever virus by adult mosquitos after ingestion of virus as larvae.. Am J Trop Med Hyg.

[57] Murithi RM, Munyua P, Ithondeka PM, Macharia JM, Hightower A (2010). Rift Valley fever in Kenya: history of epizootics and identification of vulnerable districts.. Epidemiol Infect.

[58] Evans A, Gakuya F, Paweska JT, Rostal M, Akoolo L (2008). Prevalence of antibodies against Rift Valley fever virus in Kenyan wildlife.. Epidemiol Infect.

[59] Paweska JT, van Vuren PJ, Kemp A, Buss P, Bengis RG (2008). Recombinant nucleocapsid-based ELISA for detection of IgG antibody to Rift Valley fever virus in African buffalo.. Vet Microbiol.

[60] Youssef BZ (2009). The potential role of pigs in the enzootic cycle of rift valley Fever at alexandria governorate, egyp.. J Egypt Public Health Assoc.

[61] Youssef BZ, Donia HA (2002). The potential role of rattus rattus in enzootic cycle of Rift Valley Fever in Egypt 2-application of reverse transcriptase polymerase chain reaction (RT-PCR) in blood samples of Rattus rattus.. J Egypt Public Health Assoc.

[62] Domenech J, Lubroth J, Eddi C, Martin V, Roger F (2006). Regional and international approaches on prevention and control of animal transboundary and emerging diseases.. Ann N Y Acad Sci.

[63] Aklilu Y, Irungu P, Reda A (2002). An audit of the livestock marketing status in Kenya, Ethiopia and Sudan..

[64] Rich KM, Wanyoike F (2010). An assessment of the regional and national socio-economic impacts of the 2007 Rift Valley fever outbreak in Kenya.. Am J Trop Med Hyg.

[65] Dehove A (2010). One world, one health.. Transbound Emerg Dis.

[66] Jost CC, Nzietchueng S, Kihu S, Bett B, Njogu G (2010). Epidemiological assessment of the Rift Valley fever outbreak in Kenya and Tanzania in 2006 and 2007.. Am J Trop Med Hyg.

[67] Consultative group for RVF decision support (2010). Decision-support tool for prevention and control of Rift Valley fever epizootics in the Greater Horn of Africa.. Am J Trop Med Hyg.

[68] Abdel Aziz MA (2008). Rift Valley Fever: The story unfolds….. Sudanese Journal of Public Health.

[69] LaBeaud AD, Kazura JW, King CH (2010). Advances in Rift Valley fever research: insights for disease prevention.. Curr Opin Infect Dis.

[70] Bouloy M, Flick R (2009). Reverse genetics technology for Rift Valley fever virus: current and future applications for the development of therapeutics and vaccines.. Antiviral Res.

[71] Naslund J, Lagerqvist N, Habjan M, Lundkvist A, Evander M (2009). Vaccination with virus-like particles protects mice from lethal infection of Rift Valley Fever Virus.. Virology.

[72] Kortekaas J, de Boer SM, Kant J, Vloet RP, Antonis AF (2010). Rift Valley fever virus immunity provided by a paramyxovirus vaccine vector.. Vaccine.

[73] OIE (2007). Rift Valley fever, Sudan..

[74] WHO (2008). Rift Valley fever in Sudan - update 5, January 22..

